# Contralateral translaminar endoscopic approach for highly down-migrated lumbar disc herniation using percutaneous biportal endoscopic surgery: Original research

**DOI:** 10.1186/s12893-024-02348-9

**Published:** 2024-02-16

**Authors:** Wei Cheng, Wenshuo Gao, Chengyue Zhu, Rongxue Shao, Dong Wang, Hao Pan, Wei Zhang

**Affiliations:** Department of Orthopaedics, Hangzhou TCM Hospital affiliated to Zhejiang Chinese Medical University, Tiyuchang Road NO. 453, Hangzhou, 310007 China

**Keywords:** Disc herniation, Percutaneous discetomy, Endoscopic

## Abstract

**Objective:**

Unilateral biportal endoscopy (UBE)is a minimally invasive spine surgery with reduced traumatization of the posterior lumbar ligament and muscular structures. This study reports contralateral translaminar approach with UBE for highly down-migrated lumbar disc herniation (LDH).

**Methods:**

Data of 32 patients with highly down-migrated LDH treated using UBE at our center from January 2020 to July 2022 were retrospectively analyzed. The operation time and perioperative complications were recorded, and the visual analog scale (VAS) of pain was recorded to evaluate the degree of lower back and extremity pain. The Oswestry disability index (ODI) was used to evaluate lumbar spine function. The modified MacNab score was used to evaluate clinical efficacy.

**Results:**

All patients successfully underwent the operation, with a time range from 47 to 65 min and an average operation time of 56.09 ± 5.11 min. Overall, 17 and 15 were males and females, respectively, with ages ranging from 34 to 72 years and an average age of 56 ± 7.89 years. The postoperative follow-up period was 12–18 months, with an average of 14.9 ± 2.3 months. The postoperative lower back VAS pain score and ODI were statistically significant compared with preoperatively (*P* < 0.05). At the final follow-up, according to the modified Macnab criteria, 90.6% of cases were classified as good or excellent.

**Conclusion:**

UBE treatment of highly down-migrated LDH through the contralateral translaminar approach is safe and efficient. Therefore, this approach can be an efficient alternative for patients with highly downward-migrating LDH.

## Introduction

Endoscopic discectomy is a common, minimally invasive surgical method for patients with lumbar disc herniation (LDH). Endoscopic discectomy results in less surgical trauma, more bone structure preservation, and reduced postoperative recovery time than the traditional posterior laminectomy decompression (IEDL) [1]. However, percutaneous transforaminal endoscopy technology has many shortcomings; the single-channel technology provides a narrow field of vision and insufficient stereoscopic sense, and the working channel is fixed and difficult to move [2, 3]. With the expansion of the scope of clinical application of spinal endoscopy, the shortcomings of thoracoscopy in treating free disc herniation have become more obvious [4, 5]. Heo et al. first used unilateral biportal endoscopy (UBE) technology in 2017 and applied it to minimally invasive lumbar surgery [6, 7]. Compared with percutaneous endoscopic lumbar discectomy (PELD), UBE technology uses two small unilateral incisions to insert the observation and operation channels. This method can achieve effects similar to those of open surgery, considering the clear vision and simple and flexible surgical instruments. Moreover, the endoscopic contralateral approach is a minimally invasive spine surgery with reduced traumatization of the posterior lumbar ligament and muscular structures [8].

This study aimed to introduce the surgical technique of contralateral translaminar endoscopic removal of highly down-migrated LDH using a percutaneous biportal endoscopic approach and to present preliminary clinical and radiological results.

## Methods

### Materials and methods

This study was approved by the institutional ethics committee and all patients provided signed informed consent (NO.2019KY006). The procedure is performed with the informed consent of all patients. According to Lee’s classification, a herniation is considered to be a highly migrated herniation if the degree of disc migration is greater than the posterior marginal disc height measured from the level of the adjacent endplate on t2-weighted sagittal MRI images. Based on this definition, all cases in this study could be classified as highly down migrated herniation. Since January 2020, 32 patients (17 men and 15 women, mean age 56 ± 7.89 years, range 34 to 72) affected with down-migrated LDH underwent a UBE via the contralateral translaminar approach.Inclusion criteria were patients with a soft disc herniation as demonstrated by computed tomography (CT) and magnetic resonance imaging (MRI); a lack of response to extensive conservative treatments. The exclusion criteria were non-downward migrated LDH, lumbar spinal stenosis, lumbar instability, and lumbar spinal infection or tumor.

### Surgical technique

**Fig. 1:**
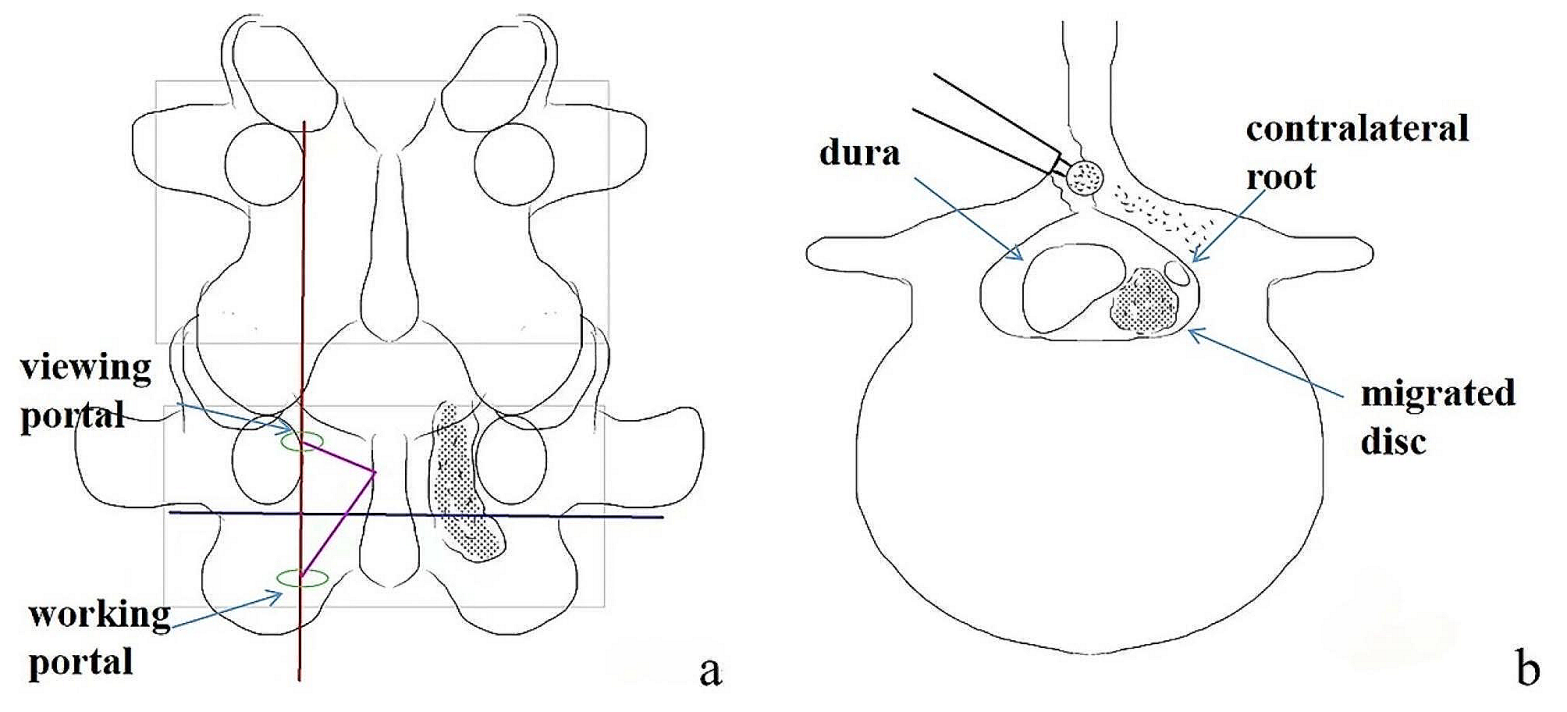
Schematic representation of the location of the portals **(a)**. The base of the spinous process was partially resected to make a working space for the endoscope. **(b)**

Under general anesthesia, the patient was placed in a prone position on an operating frame. The target level of the procedure was assessed using a C-arm fluoroscope pre- and intra-operatively. Firstly, the viewing and working ports were created on the medial border of the pedicle line on the anteroposterior view: an endoscopic port was created 15 mm above, and a working port below the pedicle line (Fig. 1a). Secondly, a drill was used to remove the middle portions of the ipsilateral laminar and the base of spinous process (Fig. 1b). The lower part of the superior lamina was removed until the proximal insertion of the LF was exposed. After the proximal and distal ends of the ipsilateral ligamentum flavum were exposed, the contralateral sublaminar area was decompressed to explore the inferior and superior sides of the contralateral pedicle. Thirdly, the ipsilateral and contralateral sides of the LF were removed to expose the dura, contralateral nerve roots, and nucleus pulposus. The ruptured fragments were then removed using a Kerrison punch and pituitary forceps. The tilting endoscope can easily reveal the contralateral sublaminar space without tilting the patient. Finally, decompression of the nerve root was confirmed, a drain was inserted, and the surgical incisions were closed.

### Clinical evaluation

The preoperative information, perioperative, and postoperative data were collected and evaluated. The clinical outcomes were evaluated by collecting visual analog scale (VAS) questionnaire answers on back and leg pain intensity and Oswestry disability index (ODI) answers for disability preoperatively and at 1 day, 6 months, and 12 months postoperatively. Patient satisfaction with clinical outcomes was assessed using the modified MacNab criteria, which includes the following four grades: excellent, good, fair, and poor, with excellent and good recognized as clinically satisfactory. Leg and back pain and neurological function were evaluated using the VAS and ODI, respectively.

### Radiological assessment

**Fig. 2:**
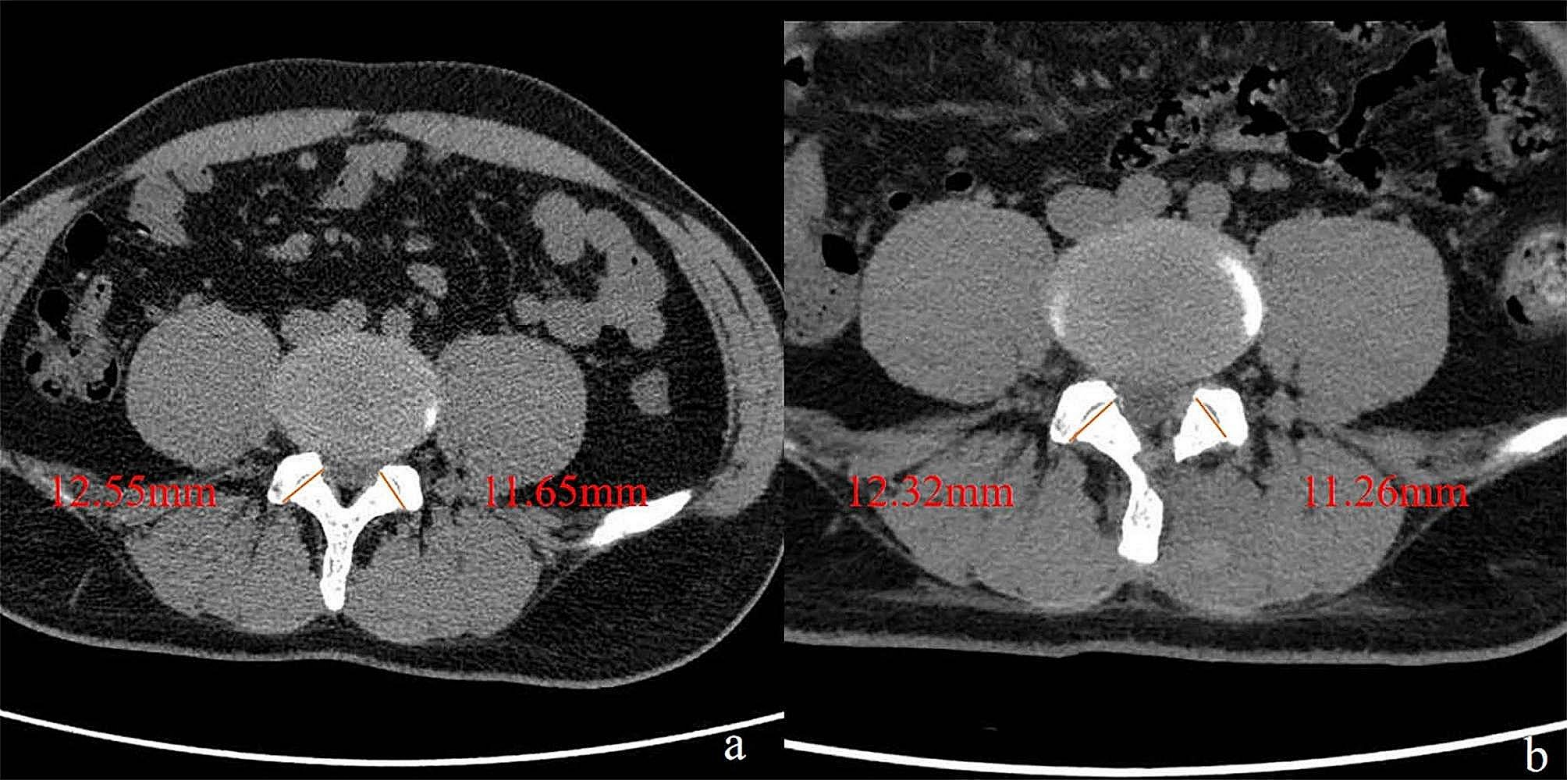
Measuring the length of facet joint plane and calculated the ratio of ipsilateral/contralateral the length of facet joint plane in preoperative **(a)** and postoperative axial CT images **(b)**. Preoperative ratio is 0.93 (= 11.65 [contralateral]:12.55 [ipsilateral]). Postoperative ratio is 0.91 (= 11.26:12.32). Reduction rate is 2.15% ([0.93 − 0.91/0.93] × 100)

The development of segmental instability or the progression of medically induced vertebral slippage was observed during the follow-up period. Postoperative MRI was performed three days after surgery to check for postoperative complications, such as inadequate nerve decompression, residual disc, facet joint invasion, and postoperative hematoma. We measured the facet joint facet length in preoperative and postoperative CT images and calculated the ratio of ipsilateral/contralateral facet joint facet length (Fig. 2a and b). The percentage of the difference between the preoperative ratio of the facet joint surface and the postoperative ratio was studied as the reduction rate of the facet joint.

### Statistical analysis

Data were recorded using Microsoft Excel 2013, and the results are reported as the mean and standard deviation. IBM SPSS Statistics 20 was used for the data analysis. Clinical outcomes, based on the modified MacNab criteria, were categorized as excellent, good, fair, and poor. The differences in pre-and post-operative VAS or ODI scores at different time points were calculated statistically using repeated measures analysis of variance. Paired and independent sample tests were used to compare the treatment values, and statistical significance was considered at *P* < 0.05.

## Results

### Clinical outcomes

This study included 32 patients (17 men and 15 women; mean age 56 ± 7.77 years ranging from 34 to 72 years). All patients had no neurologic deficit on preoperative examination. In total, 5, 4, 13, and 10 cases had L2/3, L3/4, L4/5, and L5/S1 disc herniation, respectively. The average operation time was 56.09 ± 5.11 min. The mean hospitalization time was 5.29 ± 2.11 days. Table 1 summarizes the demographic characteristics of the participants.

**Table 1:** Base demographic and clinical characteristics

Variables	Outcomes
Gender (male: female) (*n*)	17: 15
Age (mean ± SD) (years)	56 ± 7.77
Herniated segment (*n*)	
L2/3	5
L3/4	4
L4/5	13
L5/S1	10
Operation time (mean ± SD) (minutes)	56.09 ± 5.11
Hospital stay (mean ± SD) (days)	5.29 ± 2.11
Complications (*n*)	
Postoperative dysesthesia	1

Additionally, 32 patients with highly downward-migrated LDH were successfully treated, with 21 excellent and 8 good outcomes per the MacNab criteria (Table 2). The average ODI scores reduced significantly from 69.75 ± 9.88 preoperatively to 17.47 ± 4.76 at 1 day postoperatively, 15.78 ± 4.33 at 6 months postoperatively, and 14.47 ± 3.46 at 12 months postoperatively (*P* < 0.05) (Table 3). The 10-point VAS of low back and radicular pain improved from 7.25 ± 1.46 and 7.69 ± 1.47, respectively, preoperatively to 2.78 ± 0.66 and 2.41 ± 0.71, respectively, at the final follow-up (Table 3). Postoperative magnetic resonance imaging (MRI) revealed the complete removal of distally and inferiorly migrated fragments in all 32 patients. During the follow-up period, none of the patients relapsed or were readmitted to the hospital. One patient had early postoperative dysesthesia of the traversing root that was satisfactorily resolved with neurotrophic drugs for 2 weeks. No occurrence of nerve root injury or dura tear was observed.

**Table 2:** Modifed MacNab criteria

	Excellent	Good	Fair	Poor
**Patients (** ***n*** **)**	21	8	3	0
**Percentage (%)**	65.6	25	9.4	0

**Table 3:** Detailed results of clinical outcomes

Variables	Pre-op	1d post-op	6 m post-op	12 m post-op	Statistic F	*p*
VAS back pain	7.25 ± 1.46	3.44 ± 0.95	2.88 ± 0.80	2.78 ± 0.66	141.8	0.00
VAS leg pain	7.69 ± 1.47	2.81 ± 0.74	2.53 ± 0.72	2.41 ± 0.71	224.9	0.00
ODI scores	69.75 ± 9.88	17.47 ± 4.76	15.78 ± 4.33	14.47 ± 3.46	615.1	0.00

VAS Visual analog scale, ODI Oswestry disability index

### Radiological outcomes

**Fig. 3:**
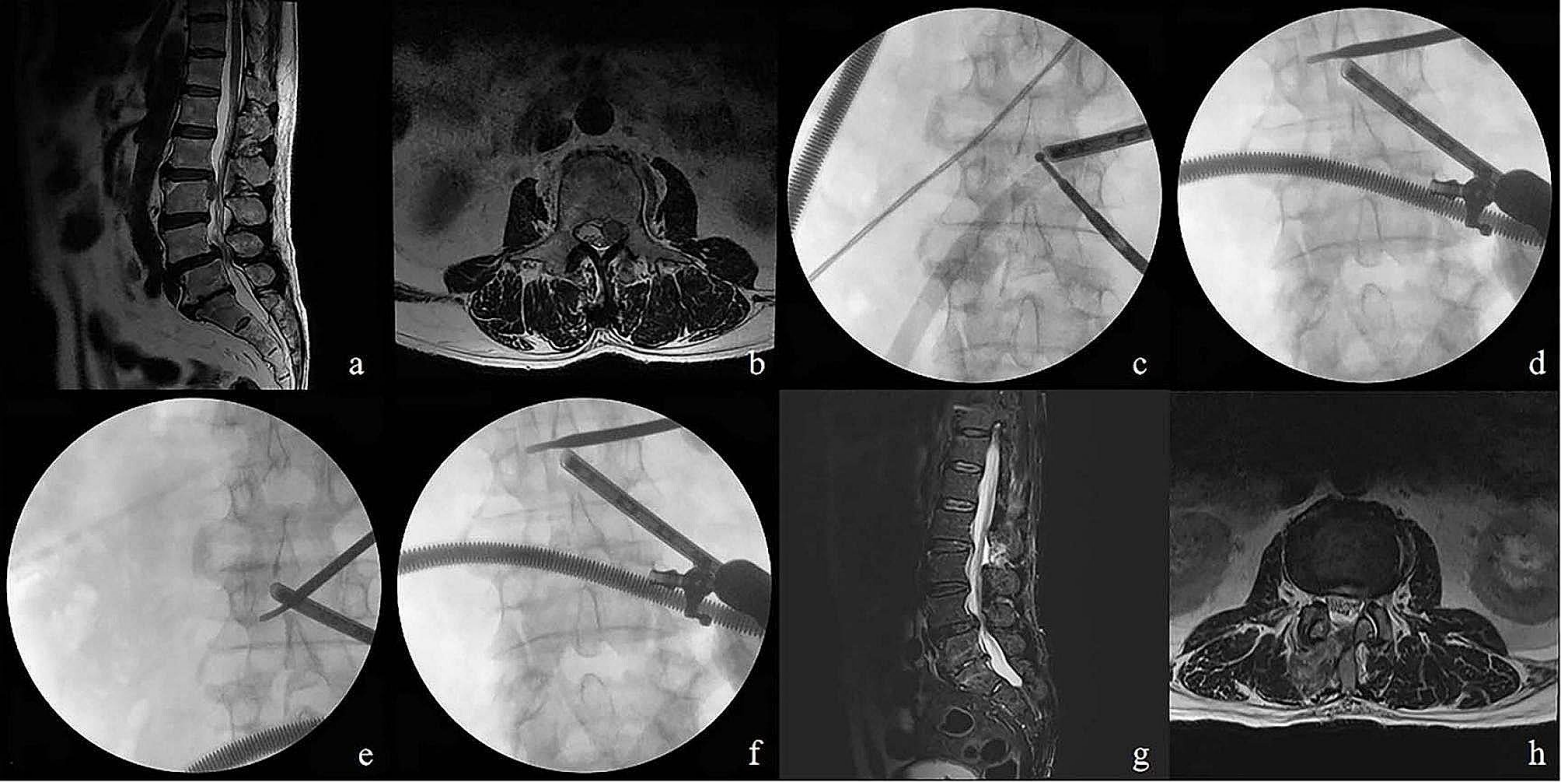
L2/3 down migrated disc shown in preoperative T2-weighted sagittal magnetic resonance imaging in sagittal **(a)** and axial views **(b)**. Desired initial tagert portal position in X-rays **(c)**. X-ray confirms that the tool is operated to the opposite side **(d)**. Intraoperative fluoroscopicimage confirming inferior **(e)** and superior **(f)** of pedicle and complete decompression of traversing nerve root. Complete removal of ruptured disc without remnant disc material in postoperative MR images in sagittal **(g)** and axial views **(h)**

The ruptured disc material was successfully removed and confirmed by postoperative MRI (Fig. 3). no significant small joint invasion or postoperative epidural hematoma was observed on the MRI scan. The mean preoperative ipsilateral/contralateral subtalar articular surface ratio was 0.97 ± 0.028 (1.0-0.93) and the mean postoperative ratio was 0.96 ± 0.025 (1.04–0.91). There was no statistically significant difference between the preoperative ratio and the postoperative value (*p* > 0.05). The rate of reduction of the facet joint.

plane was approximately 1.03%. During the follow-up period, no new segmental instability or spondylolisthesis was observed on plain flexion and extension films.

### Illustrative cases

**Fig. 4:**
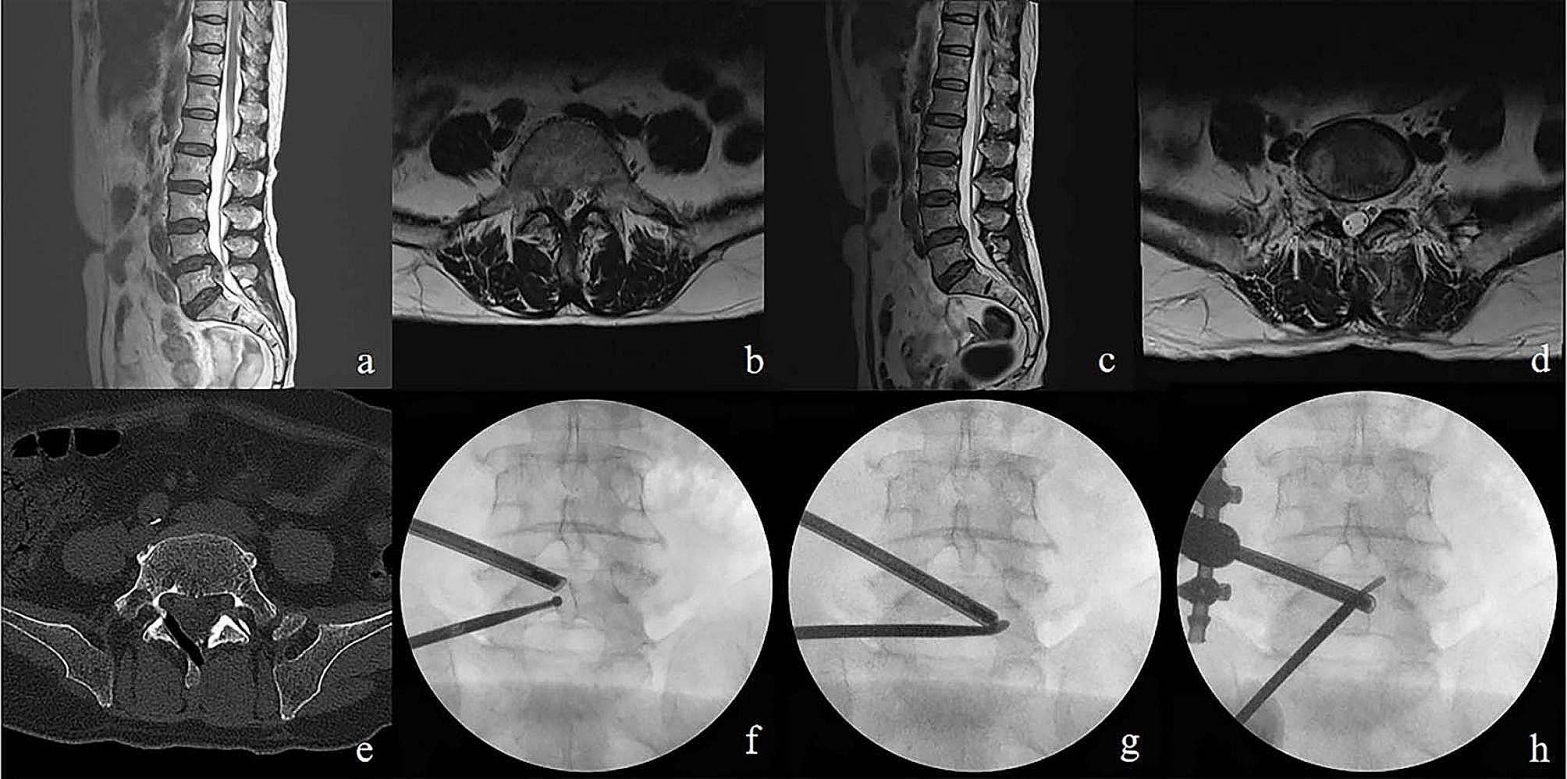
L4/5 down migrated disc shown in preoperative T2-weighted sagittal magnetic resonance imaging in sagittal **(a)** and axial views **(b)**. Complete removal of ruptured disc without remnant disc material in postoperative MR images in sagittal **(c)** and axial views **(d)**. Keyhole in contralateral spinolaminar junction without violation of facet joint shown in postoperative CT image **(e)**. Desired initial tagert portal position in X-rays **(f)**. Intraoperative fluoroscopicimage confirming inferior **(g)** and superior **(h)** of pedicle and complete decompression of traversing nerve root

**Fig. 5:**
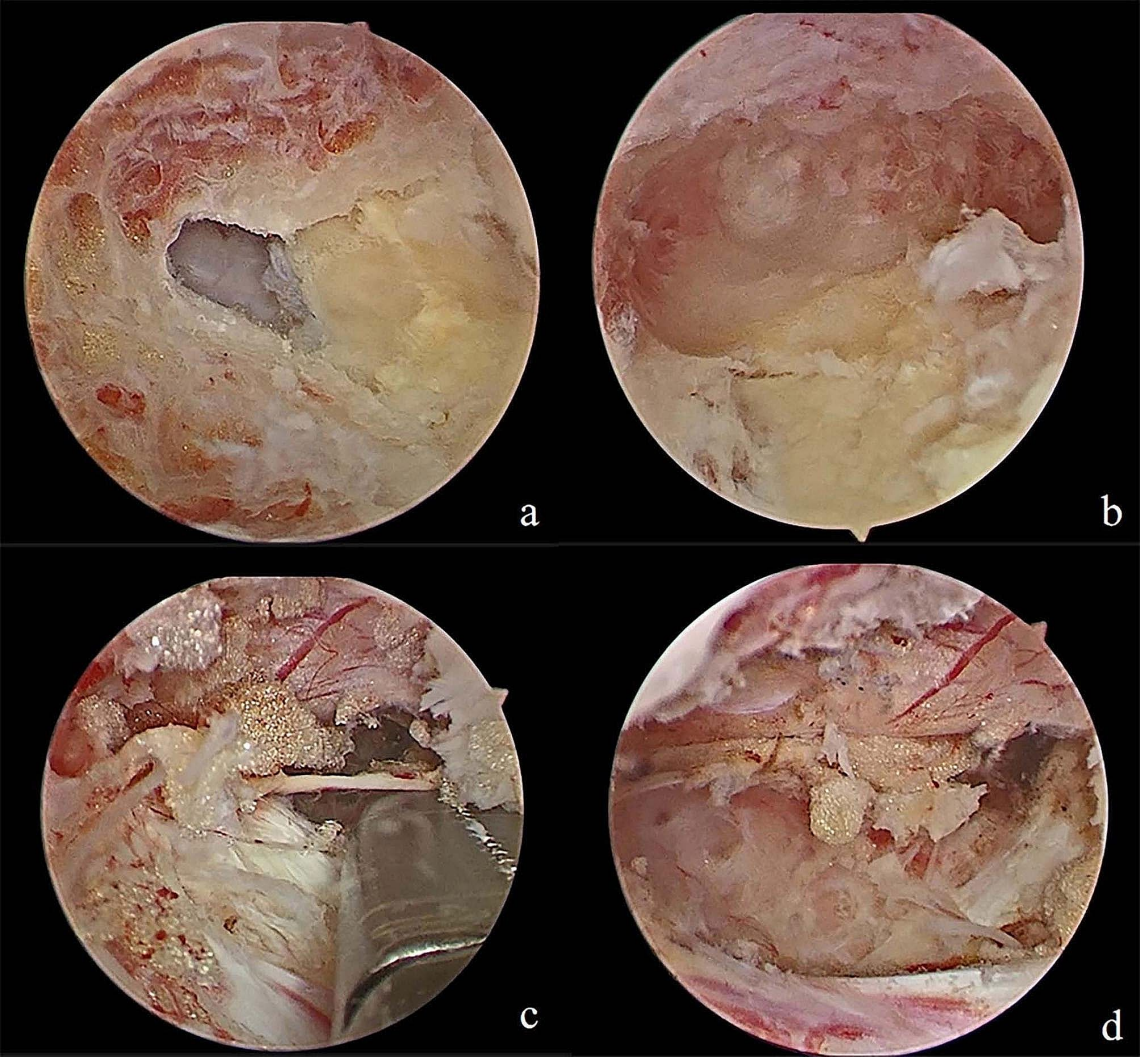
Intraoperative endoscopic images of right approach. **(a)** Intraoperative endoscopic images of right approach. After making keyhole, central fissure of ligamentum flavum was identified at first **(b)**. Removal of ruptured disc material can be performed safely using specialized hand-made retractor with pituitary forceps during retraction of traversing nerve root **(c)**. The procedure is done when the full decompression of the dura and nerve roots are confirmed **(d)**

**Fig. 6:**
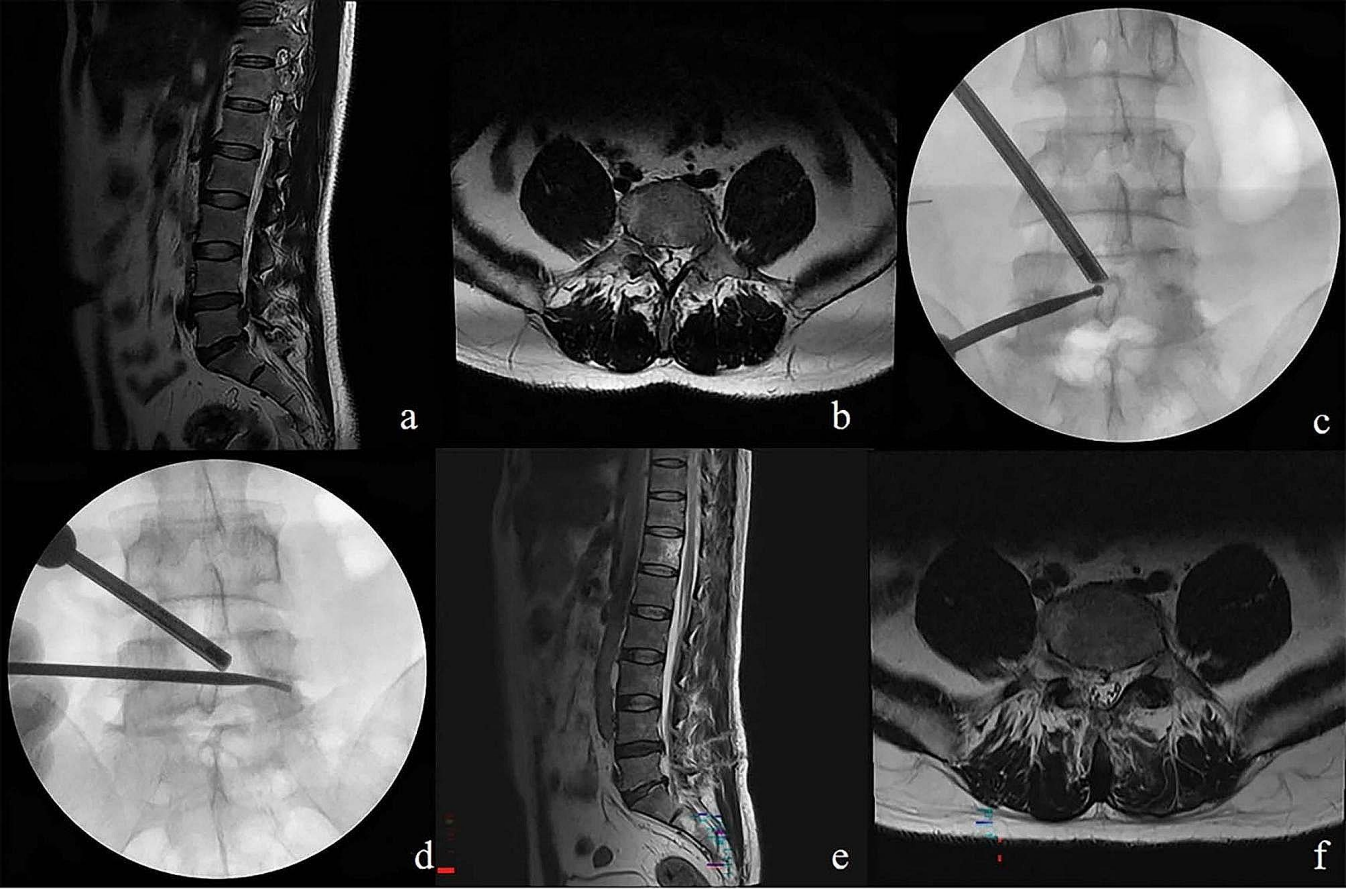
L4/5 down migrated disc shown in preoperative T2-weighted sagittal magnetic resonance imaging in sagittal **(a)** and axial views **(b)**. Desired initial tagert portal position in X-rays **(c)**. Intraoperative fluoroscopicimage confirming inferior of pedicle and complete decompression of traversing nerve root **(d)**. Complete removal of ruptured disc without remnant disc material in postoperative MR images in sagittal **(e)** and axial views **(f)**

**Fig. 7:**
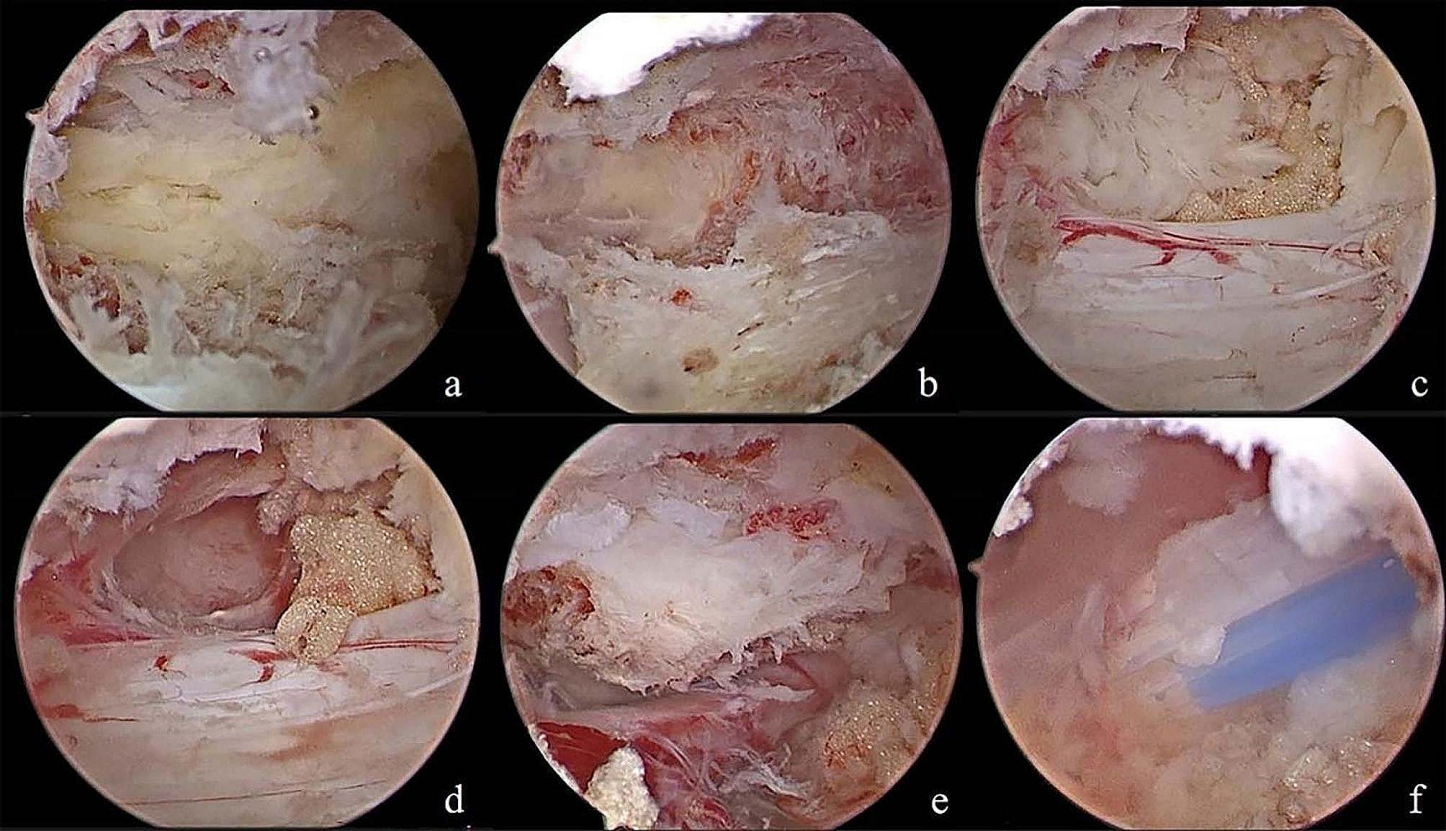
Intraoperative endoscopic images of right approach. **(a)** Intraoperative endoscopic images of right approach. After making keyhole, central fissure of ligamentum flavum was identified at first. **(b)** The contralateral translaminar portal was established. **(c)** Exposed contralateral migrated nucleus pulposus tissue. **(d)** Removal of ruptured disc material can be performed safely using specialized hand-made retractor with pituitary forceps during retraction of traversing nerve root. **(e)** The axilla and shoulder of the contralateral nerve root was decompressed. **(f)** A drain was inserted into the working portal

Fig. 3a-h presents the pre-and postoperative images and intraoperative fluoroscopic views of a 72-year-old male patient treated with the L2/3 contralateral translaminar UBE approach. Two illustrative cases of a very highly down-migrated disc are presented in Figs. 3–7.

## Discussion

Migrated LDH is common in clinical practice, accounting for 35–72% of LDH, and highly migrated LDH for 13–25%; downstream separation is more common than upstream dissociation [9, 10]. Open surgery requires dissection of the paravertebral muscle and removal of the lamina and articular process, which may result in spinal motor segment instability and intractable lower back pain. Percutaneous spinal endoscopic techniques have the same clinical efficacy as traditional open surgery with fewer complications. With the continuous progress of technology and instruments, PELD has become possible for the treatment of highly-down migrated lumbar disc herniation. Ying et al. [11] introduced superior border of inferior pedicle approach for down migrated intracanal disc herniations via PELD, and their VAS and ODI scores were significantly improved at the end of follow-up. However, in this study, some patients had postoperative complications, especially incomplete removal of fragments, which required another open operation. And it requires a long learning curve to mature the technology. In addition, some scholars have used the interlaminar approach for the treatment of high grade down migrated L4/5 disc [12], but this approach has a high risk of dural injury and is prone to cerebrospinal fluid leakage when there is no protection of the yellow ligament after the bite of the yellow ligament. Consequently, facet wear and incomplete fragmentation are risks, leading to segmental instability that requires subsequent revision with open surgery in some patients [13, 14]. The limited visual field, insufficient exposure, and difficulty in grasping microscopic disc fragments are the main reasons for the failure of traditional percutaneous endoscopic lumbar nucleus pulposus removal techniques in highly relapse-free LDH treatment. Therefore, some clinicians have used the UBE technology to treat degenerative diseases of the lumbar spine [13]^,^ [14]. The UBE procedure is similar to that of conventional microscopic lumbar discectomy, with the surgical anatomy visualized by magnifying the pathological lesion with a 4 mm endoscope and rinsing the surgical area with continuous saline [15]. Moreover, all microsurgical instruments, such as high-speed grinding drills and Kerrison laminar bone forceps, can also be used for UBE surgery, greatly improving the efficiency [16].

The UBE technique combines the merits of standard open discectomy and endoscopic discectomy. The surgical procedure for UBE is similar to a traditional open discectomy; the range of the approach can be widened with an inclined introduction and pivoting motion of the endoscope through the contralateral translaminar window. Articular joint injury during decompression has been reported in patients with upper lumbar lesions, spinal stenosis, and sagittal facet joint morphology. To avoid iatrogenic instability of the lumbar spine caused by the facet joint invasion after laminectomy, some scholars have used the contralateral UBE approach to treat LDH, with good clinical and surgical outcomes [15, 16]. Jung Hoon Park showed that the facet reduction rate of the contralateral UBE approach is approximately 4.9%, which is lower than the articular surface resection rate of the previously reported ipsilateral approach [17]. Indeed, our postoperative follow-up results did not find associated iatrogenic instability.

The UBE procedure begins at the junction of the spinous process and lamina without attached muscle or vascular supply. Preservation of the paravertebral muscles and facet joints is the most important consideration in non-fusion endoscopic spinal surgery, particularly on the pathological side. Patients with highly down-migrated LDH need to remove the lamina and bite off the sublaminal ligament flavum to reveal the compressed nerve root and nucleus pulposus tissue. By drilling a bony tunnel in the lamina, this approach may allow the working cannula to directly target the highly down-migrated disc herniation. In the contralateral approach, the paravertebral muscles on the pathological side and the articular process are less damaged. In a study by Ahn [18], MRI scans immediately after ULBD showed significant changes in ipsilateral and contralateral muscle signals related to the time of surgery. At two weeks follow-up, the signal intensity ratio (SIR) of the ipsilateral and contralateral polyfissures increased by 52% and 24.7%, respectively. As the multifidus muscle is innervated by a single segment of the dorsomedial branch of the spinal nerve, the risk of damage is higher with devices in the lateral crypt and intervertebral foramen area in the ipsilateral approach; the contralateral approach can effectively reduce postoperative multifidus loss innervation [19].

The contralateral translaminar approach has several advantages. First, the working cannula is passed through a bony tunnel but not through soft tissues to reach the herniation, potentially avoiding injury to the adjacent soft tissue during the process of puncture and construction of the working cannula. Second, cranial and caudal exploration can be performed to completely remove the fragments, preventing the residual nucleus pulposus tissue from affecting the postoperative efficacy. Third, Adequate sublaminar space allows the free nucleus pulposus tissue to be fully exposed, avoiding extensive resection of the lamina and facet joints, which can lead to postoperative instability and other complications. This approach also has some limitations. First, there may be a learning curve for this technique because the contralateral translaminar approach is not familiar. Surgeons who are familiar with microendoscopic bilateral decompression via the unilateral approach would likely be able to perform this method efficiently and easily. Second, grinding the base of the spinous process to avoid being too horizontal, otherwise, the spinous process may be fractured. Third, sufficient sublaminar space should be created for free use of spinal endoscopes and instruments, and dural tears may occur during the enlarging of the bottom of the bone tunnel without yellow ligament cover.

Our results showed significant improvement in postoperative clinical outcomes in patients with highly down-migrated LDH treated with a contralateral translaminar approach via UBE. Our cases demonstrated no neurological complications after surgery, such as poor postoperative efficacy due to residual nucleus pulposus tissue.

The study had some limitations. This was a retrospective study with a small sample size and short follow-up period. In addition, due to the nature of retrospective studies, selection bias appears to be an intrinsic factor in patient preference, and surgeon experience may influence outcomes. Detailed prospective trials using a larger cohort that compares UBE with other techniques, such as conventional surgery, are needed for a deeper analysis of this topic. Nevertheless, we wanted to share our own experience with highly down migrated LDH.

In conclusion, we completely removed highly down-migrated LDH by performing percutaneous biportal endoscopic surgery via the contralateral translaminar approach. Therefore, this approach may be a viable alternative for down-migrated LDH treatment, with minimal iatrogenic facet violation and traumatization of the posterior muscle and ligamentous structures.

## Data Availability

The datasets generated during and analyzed during the current study are not publicly available, but are available from the corresponding author on reasonable request.
